# A review of removal of per- and polyfluoroalkyl substances using metal–organic framework-based nanoadsorbents

**DOI:** 10.1186/s40580-025-00521-1

**Published:** 2025-11-06

**Authors:** Doorae Lee, Byung-Moon Jun, Jungyeon Park, Min Jang, Seunghyun Lee, Shane A. Snyder, Chang Min Park, Yeomin Yoon

**Affiliations:** 1 Department of Chemical and Environmental Engineering, University of Arizona, Tucson, AZ 85721 USA; 2 Department of Environmental Science & Engineering, Ewha Womans University, 52 Ewhayeodae-Gil, Seodaemun-Gu, Seoul, 03760 Republic of Korea; 3 Department of Environmental Science and Engineering, Kyung Hee University, 1732 Deogyeong-daero, Giheung-Gu, Yongin-Si, Gyeonggi-Do 17104 Republic of Korea; 4 Korea International School, Jeju Campus, 34 Global Edu-Ro 260Beon-Gil, Daejeong-Eup, Seogwipo-Si, Jeju-do, 63644 Republic of Korea; 5 Department of Environmental Engineering, Kwangwoon University, 447-1 Wolgye-Dongong Nowon-Gu, Seoul, Republic of Korea; 6 Department of Applied Chemistry, Hanyang University ERICA, Ansan, 15588 Republic of Korea; 7 Center for Bionano Intelligence Education and Research, Hanyang University ERICA, Ansan, 15588 Republic of Korea; 8 Department of Energy and Bio Sciences, Hanyang University ERICA, Ansan, 15588 Republic of Korea; 9 School of Civil and Environmental Engineering, College of Engineering, Georgia Institute of Technology, 311 Ferst Dr NW, Atlanta, GA 30332 USA; 10 Department of Environmental Engineering, Kyungpook National University, 80 Daehak-Ro, Buk-Gu, Daegu, 41566 Republic of Korea

**Keywords:** Adsorption, Perfluorooctanoic acid, Perfluorooctanesulfonic acid, Metal–organic frameworks, Adsorption, Water treatment

## Abstract

Per- and polyfluoroalkyl substances (PFASs) are a category of extremely persistent environmental pollutants. Metal–organic frameworks (MOFs) have appeared as promising adsorbents for PFAS removal due to their large surface area, tunable porosity, and versatile surface chemistry, which are among the numerous treatment technologies available. This review critically evaluates current developments in the design, fabrication, and application of MOF-based (nano)materials for the adsorption of PFAS in aqueous environments. The adsorption efficacies of MOFs (e.g., pore size, surface charge, and functional groups) and PFASs (e.g., chain length, head group functionality, and polarity) are significantly influenced by their physicochemical properties. The selective and efficient removal of PFASs is governed by the interaction mechanisms such as electrostatic attraction, hydrophobic interactions, H-bonding, and Lewis acid–base coordination. In addition, the adsorption efficacy is significantly influenced by water quality conditions, including pH, ionic strength, background ions, and natural organic matter. Functionalized MOFs (e.g., those with amine, fluorinated, or hydrophobic groups) exhibit resilience to interference, although these factors can sometimes hinder their removal. Both experimental and computational studies have provided valuable mechanistic insights into the rational design of MOFs with improved selectivity and capacity. In addition, this review identifies critical challenges and future perspectives, such as the necessity of standard performance testing under realistic water matrices; the development of scalable, stable, and regenerable MOFs; and their integration into life-cycle assessment and toxicity evaluation.

**Graphical abstract:**

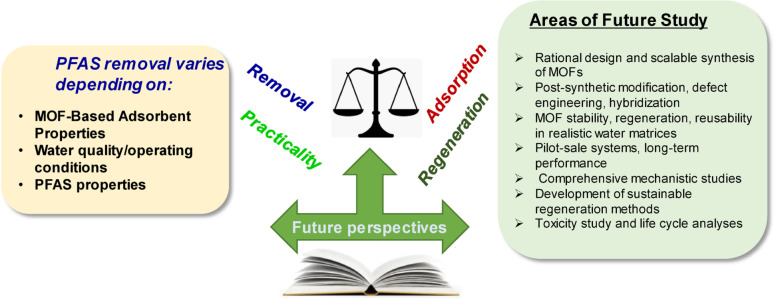

## Introduction

Per- and polyfluoroalkyl substances (PFASs) are artificial chemicals characterized by entirely or incompletely fluorinated carbon chains. They are highly persistent and widely used in a range of consumer, food-related, and specialized products, including non-stick cookware, water/stain repellents, shampoos/conditioners, cleaning agents, fast food wrappers, grease-resistant paper, aqueous film-forming foams, paints/sealants, and food packaging [1, 2]. However, their widespread application has resulted in the broad occurrence of PFASs in water bodies [3]. In particular, long-chain PFASs, including perfluorooctane sulfonic acid (PFOS) and perfluorooctanoic acid (PFOA), have very low human health advisory concentrations (ng/L to µg/L) [4]. Recently, the U.S. Environmental Protection Agency confirmed the first National Primary Drinking Water Regulation for PFOA and PFOS, setting the officially enforceable maximum contaminant level at 4 ng/L, which is among the lowest concentrations achievable by standardized analytical methods [5].

PFASs have thousands of distinct molecular structures that differ in the number of extremely powerful/stable carbon–fluorine bonds, various functional groups, anionic/non-ionic/zwitterionic forms, and the presence of heteroatoms [6], which pose significant challenges for their removal during water and wastewater treatment processes. However, various traditional and innovative water/wastewater treatment methods have been studied to assess effectiveness for PFAS removal, including coagulation/flocculation/sedimentation [7], granular activated carbon [8], powdered activated carbon [9], metal–organic frameworks (MOFs) [10], ion exchange resins [11], reverse osmosis membrane/nanofiltration [12], ultraviolet photocatalytic degradation [13], ozonation/H_2_O_2_ [14], thermal oxidation [15], plasma treatment [16], and bioremediation [17].

Because of their large surface area, adjustable porosity, and changeable surface chemistry, MOFs have demonstrated great promise as materials for the adsorptive removal of numerous PFASs from water [18]. The adsorption capability of MOFs can be significantly improved by surface modification and functionalization, including the addition of amine groups, quaternary ammonium groups, fluorophilic functional groups, and hydrophobic alkyl chains [19]. In particular, the synergistic integration of MOFs with metal-based adsorbents (e.g., Fe_3_O_4_, Mg/Al oxides, FeOOH, and zero-valent iron) has been shown effective for the removal of PFASs [18, 20].

Recently, numerous studies have evaluated the removal of PFASs using advanced porous sorbents [1], covalent organic frameworks [18], activated carbon/biochar/polymers/resins [21], functional framework materials [22], and MOFs [20, 23, 24]. However, those evaluating MOFs have primarily focused on the design strategies of different MOFs, their characteristics, and their roles. Therefore, a comprehensive and systematic review is essential for evaluating the effectiveness of MOF-based nanoadsorbents for the removal of various PFASs from water. Their physicochemical characteristics, such as structure, surface area, functional groups, hydrophobicity/hydrophobicity, surface charge, and pKa, as well as performance-influencing environmental parameters, such as pH, background anions/cations, and organic matter, were thoroughly examined. Accordingly, this review critically examines the current advances in the application of MOF-based nanoadsorbents for PFAS removal. In addition, potential future research directions are outlined to understand existing knowledge gaps and guide further development in this field.

## Preparation of various MOF-based nanoadsorbents

Solvothermal synthesis is one of the most frequently used processes for preparing MOFs. It involves the interaction of metal ions and organic linkers in a solvent at elevated temperatures (typically 100–250 °C) in a sealed vessel (such as an autoclave) [25]. The solvent dissolves the precursors and supports crystal formation over time under controlled temperature and pressure conditions. In a previous study, Cu-based MOF was prepared by dissolving trimesic acid in ethanol and copper nitrate trihydrate in water to fabricate a Cu-based MOF with amine-functionalized SiO_2_ nanoparticles (A-CMOF) [26]. These solutions were combined, stirred for 45 min, moved into a sealed stainless-steel autoclave, and heated at 120 °C for 12 h. After the reaction, the solid product was cleaned with ethanol and water and dehumidified at 150 °C for 24 h. Separately, NH_2_-functionalized silica nanoparticles were synthesized by mixing ethanol, water, ammonia, and SiO_2_ nanoparticles, followed by slow addition of 3-aminopropyl triethoxysilane over 6 h. After centrifugation and washing, the particles were dried at 60 °C. For the modified MOF (A-CMOF), trimesic acid was dissolved in a N,N-dimethylformamide-ethanol mixture, and NH_2_-SiO_2_ nanoparticles were added. Copper nitrate in water was then introduced, and the mixture was stirred at 70 °C for 4 h before undergoing the same solvothermal treatment. The final product, A-CMOF, was isolated as a white powder and preserved for further use [26].

The MOF PCN-222 was fabricated via solvothermal synthesis with post-synthetic solvent exchange and activation, which was subsequently employed to remove PFOS from water [27]. Here, zirconium tetroxide and tetrakis(4-carboxyphenyl)porphyrin were dissolved in dimethylformamide containing trifluoroacetic acid (TA) in a glass vial. The solution was sonicated for 15 min and stirred in an oil bath at 120 °C for 10 min before being cooled to 20 °C. Red needle-like crystals of PCN-222 were collected by centrifugation at 10,000 rpm for 5 min. The product was cleaned several times with dimethylformamide and incubated overnight. Dimethylformamide was replaced daily for 2 d, followed by methanol exchange for 3 d, with daily solvent replacement. Lastly, the solid was cleaned with acetone and dried under a vacuum at 150 °C overnight [27].

Highly defective UiO-66 MOFs were employed as effective adsorbents for removing PFOS from concentrated solutions [10]. The concentration of structural defects in UiO-66 was controlled by adjusting the volume percentage of concentrated HCl during the solvothermal fabrication. UiO-66@X materials (where X denotes the vol% of HCl: 10, 25, or 50) were prepared by dissolving 3.2 mM of ZrCl_4_ in dimethylformamide to a total volume of 50 mL, with the appropriate volume of HCl. The mixture was sonicated at room temperature in a water bath until ZrCl_4_ completely dissolved. Subsequently, 3.2 mM of terephthalic acid and the remaining HCl were added, and the volume was adjusted to 100 mL with dimethylformamide. After sonication for 30 min, the solution was divided equally into 40 mL vials, sealed, and heated at 120 °C for 24 h. The subsequent solids were cleaned three times with dimethylformamide, washed three times with methanol, and vacuum-filtered to remove residual solvents. For comparison, a defect-free UiO-66 MOF was fabricated using established procedures.

Various fluorinated UiO-MOFs (UiO-66, UiO-66-F_2_, and UiO-66-F_4_) were prepared via a hydrothermal process utilizing ZrO(NO_3_)_2_·xH_2_O as the metal precursor along with distinct organic ligands: terephthalic acid, 2,5-difluoroterephthalic acid, and 2,3,5,6-tetrafluoroterephthalic acid, respectively, which were used to remove one of the emerging PFASs (sodium p‑perfluorous nonenoxybenzenesulfonate, OBS) [28]. In the fabrication of UiO-66-F4, ZrO(NO_3_)_2_·xH_2_O and 2,3,5,6-tetrafluoroterephthalic acid were solubilized in ultrapure water using ultrasonication. Subsequently, acetic acid was added, and the mixture was exposed to heating in a Teflon-lined autoclave at 120 °C, after which it was cooled to 25 °C. The resultant crystals were obtained via centrifugation, rinsed three times with deionized water, twice with ethanol, and immersed in acetone for two days, with the acetone being replaced bi-daily. The final product was obtained after vacuum-drying for 24 h. UiO-66-F_2_ and UiO-66 were synthesized using 2,5-difluoroterephthalic and terephthalic acids, respectively, as chemical ligands following an identical protocol [28].

MIL-101(Cr) was fabricated using a hydrothermal technique to remove hexafluoropropylene oxide dimeric acid (GenX) [29]. In summary, 1,4-benzenedicarboxylic acid and chromium(III) nitrate nonahydrate were solubilized in deionized water and agitated for 1 h. The resulting suspension was sonicated for 30 min at ambient temperature and subsequently transferred to a Teflon-lined autoclave. The mixture was heated to 220 °C and maintained at this temperature for 20 h. Upon reaching room temperature, the precipitate was harvested by centrifugation at 4000 rpm. The solid was cleaned three times with dimethylformamide at 100 °C in an autoclave, followed by three washes with ethanol under the same conditions to eliminate excess unreacted 1,4-benzenedicarboxylic acid. The material was further dried under vacuum at 70 °C to yield MIL-101(Cr) [29].

## Removal performance of various MOF-based nanoadsorbents

### Removal based on MOF/PFAS properties and operating conditions

#### MOF or PFAS properties

Developing MOFs with optimal properties is essential for improving the PFAS adsorption capacity, selectivity, and degradation potential, as their efficacy relies on a combination of structural attributes (e.g., surface area and pore dimensions) and chemical functions (e.g., charge and hydrophobicity) [20]. The MOFs NU-1000, UiO-66, and ZIF-8 were chosen to examine the influence of their structural properties on PFAS removal from contaminated groundwater owing to their distinct pore sizes and shapes, as well as the identification of metal nodes [30]. NU-1000 demonstrated significant selectivity for 21 anionic PFASs, with a total removal capacity of 58% for anionic PFASs and nearly complete removal (99%) for seven non-ionic PFASs. UiO-66 exhibited a high removal efficiency of 95% for non-ionic PFASs, although it had somewhat insufficient selectivity for anionic PFASs, with a removal efficiency of only 2%. However, ZIF-8 exhibited minimal attraction for both anionic and non-ionic PFASs, resulting in a total removal rate of under 10%. A previous study recognized that the adsorption of anionic perfluorosulfonic acids onto NU-1000 is predominantly governed by ionic interactions [31]. The adsorption mechanism was accurately characterized as an anion exchange reaction involving the deprotonated –COOH or –SO_3_H groups of PFASs and the coordinated hydroxo ligands on the Zr_6_ nodes of NU-1000. In addition to ionic interactions, hydrophobic interactions between the fluorinated carbon chains of anionic PFASs and the hydrophobic pockets developed by the pyrene-based ligands in NU-1000 significantly enhance the overall adsorption.

The large affinity of NU-1000 for non-ionic PFASs can be clarified by three principal mechanisms: (1) according to Pearson’s hard and soft acids and bases theory, the amine groups in non-ionic PFASs (hard bases) exhibit strong interactions with the Zr_6_ nodes (hard acids) [32]; (2) hydrophobic interactions between the C and F chains and the hydrophobic pockets of NU-1000 further facilitate adsorption [33]; (3) an alternative electrostatic interaction may occur when the amine groups of PFAS deprotonate the μ_3_-bridging hydroxo groups of the Zr_6_ nodes, causing robust μ_3_−O^2−^–NH_3_^+^ electrostatic bonds [34]. For UiO-66, the equilibrium adsorption of non-ionic PFASs was obtained during the initial 10 min, which was slower than that of NU-1000 [30]. The difference in kinetics aligns with the reduced pore sizes in UiO-66, which measure roughly 0.7 nm, in contrast to the wider channels in NU-1000. The tetrahedral and octahedral cages of UiO-66 have diameters of 0.8 and 1.1 nm, respectively. [35] reported comparable kinetic constraints for UiO-66 in their investigation of selenite removal. ZIF-8 demonstrated the slowest adsorption kinetics among all the evaluated MOFs (exceeding 30 min), presumably due to its limited pore size (0.34 nm), despite the presence of moderately expansive sodalite cages (1.34 nm in diameter) [30].

The physicochemical properties of PFASs, such as chain length, functional groups, size/structure, and electronegativity/electron density, play critical roles in determining their removal efficiencies and mechanisms [18]. Grand canonical Monte Carlo simulations were conducted to evaluate the adsorption of perfluoroalkanoic acids (PFAAs) and alkanoic acids in the MOF NU-1000, considering co-adsorbed water and variations in the PFAS carbon chain length, to assess the impact of the molecular structure on the adsorption capacity [33]. The results exhibited that the total adsorption capacity decreases as the carbon chain length increases. A previous study showed that relatively higher loadings were anticipated for longer-chain PFAS, 894 mg/g for PFOA and 730 mg/g for perfluoroheptanoic acid, in contrast to the experimental values of 507 and 421 mg/g, respectively [31], indicating that the idealized model was somewhat ineffective in accounting for the pore-blocking effects observed in actual crystals. Nevertheless, the simulation accurately reflected the tendency of longer PFAAs to preferentially partition into NU-1000, even in aqueous environments. PFAAs have superior adsorption to alkanoic acids with equal chain lengths. Although their larger van der Waals radii are often anticipated to hinder packing, PFAAs surpass alkanoic acids under gravimetric loading, a phenomenon ascribed to their higher mass of fluorine atoms. This indicates that intermolecular packing and interactions, rather than solely pore volume, enhance the adsorption of PFAAs compared with that of alkanoic acids [33].

#### Operating conditions

The operational conditions significantly influence the efficiency, selectivity, and feasibility of PFAS adsorption by MOFs, including the contact time, PFAS concentration, PFAS type (long- and short-chain), and MOF dosage [18]. Liang et al. presented a novel Zr-based MOF (PCN-999), characterized by Zr_6_ clusters and biformate-bridged secondary building units. This extraordinary structural arrangement facilitates remarkable PFOA absorption, achieving 1090 mg/g; approximately 50% greater than the previously documented maximum for MOFs [36]. The numerous available coordination sites, hierarchical micro–mesoporous structure, and exceptional chemical and thermal stabilities make PCN-999 an optimal nanoscaffold for PFOA removal. The activated MOF was submerged in an aqueous PFOA solution with an initial concentration of 1000 mg/L at 20 °C for 3 d to assess its PFOA removal efficacy. ^19^F nuclear magnetic resonance measurement indicated a PFOA loading of approximately 570 mg/g, demonstrating exceptional adsorption efficacy at equilibrium within 12 h, confirming a pseudo-2nd-order kinetics model (k_2_ = 1.38 × 10⁻^3^ g/mg-h). Equilibrium adsorption isotherms were determined using original PFOA concentrations between 100 and 5000 mg/L, confirming that the Langmuir model had greater accuracy (R^2^ = 0.96) than the Freundlich model (R^2^ = 0.91), which suggested a monolayer adsorption behavior. The maximum adsorption capacity for PFOA was 1090 mg/g at an equilibrium concentration of 3900 mg/L, which is much higher than that previously documented [37–39]. Moreover, PCN-999 exhibited over 99% removal effectiveness at an initial PFOA concentration of 1000 mg/L, validating its exceptional capability for the successful cleanup of PFOA from polluted water [36].

Both typical DUT-67 and MOF-808 were modified by integrating a fluorine-containing monocarboxylic acid as a non-structural ligand into Zr_6_ clusters, specifically 2-fluorobenzoic acid (FA), 2,6-difluorobenzoic acid (DFA), or TA [40]. This technique facilitated the precise adjustment of the pore size and hydrophobicity of MOFs through the modulation of the fluorine concentration or the incorporation of larger benzoate groups. Fluorinated ligand-functionalized materials modified with FA, DFA, or TA significantly improved adsorption efficacy for long-chain PFASs (C ≥ 8, carboxylic acid analogs) within both DUT-67 and MOF-808 adsorbents, achieving uptake enhancements of up to 28% (490 mg/g) for TA@DUT-67 and 36% (1340 mg/g) for TA@MOF-808. The adsorption isotherm results for a PFOA concentration range of 100–800 mg/L aligned somewhat more effectively with the Langmuir model, leading to maximum adsorption capacities of 1580 mg/g for pristine MOF-808 and 2500 mg/g for TA@MOF-808. TA@MOF-808 achieved total (nearly 100%) elimination of PFOA at low concentrations (100 mg/L) with a small dose of MOF, indicating its significant potential for the cleanup of environmentally relevant PFASs [41]. In comparison, only 30% of pristine MOF-808 was eliminated under identical conditions.

In addition, the identical adsorbents (MOF-808 and TA@MOF-808) were assessed for their adsorption capabilities for relatively short-chain PFASs such as perfluorohexanoic acid (C_6_) and perfluorobutanoic acid (C_4_) [40]. Unlike PFOA, the adsorption of these short-chain PFAS compounds is relatively less dependent on hydrophobic interactions owing to their shortened perfluorinated chains and fewer fluorine atoms. Thus, the post-synthetic augmentation of MOF hydrophobicity is vital for enhancing PFAS binding. Immersion of the MOFs in 100 mg/L aqueous solutions of each compound demonstrated that TA@MOF-808 significantly exceeded its non-fluorinated equivalent, with increases of 66% and 33% in uptake, along with enhancements of 40% and 12% in the elimination efficiency for perfluorohexanoic acid and perfluorobutanoic acid, respectively. The adsorption capacities of 436 mg/g for perfluorohexanoic acid and 311 mg/g for perfluorobutanoic acid by TA@MOF-808 highlight its exceptional performance as a leading porous material for the efficient capture of short-chain PFASs at environmentally relevant concentrations [40].

A hybrid structure of a metal oxide immobilized on a carbon substrate, featuring both three-dimensional and two-dimensional characteristics, was fabricated from a thermally treated derivative of an iron-based MOF, H@MIL-101(Fe) [42]. This heat-treated MOF effectively removed PFOA, achieving total elimination from pure water in 5 min. In addition, six PFAS compounds, ranging from four to eight C atoms, were completely co-adsorbed from the surface water within 10 min at pH 7.0, primarily driven by hydrophobic interactions. With the addition of 300 mg/L H@MIL-101 (Fe), the removal rate of GenX declined significantly from 96.4 to 35.4% with an increase in the initial GenX concentration (1–10 mg/L). This trend highlights the importance of adsorbent dosage in optimizing PFAS removal, suggesting that the number of available adsorption sites governs the upper limit of the adsorption performance. The introduction of multilayers within the MOF pores has been proposed as a promising strategy to enhance this capacity. To further explore the adsorption behavior, a study investigated the kinetics of PFOA and GenX by reducing the adsorbent dosage and increasing the pollutant concentrations. H@MIL-101(Fe) exhibited a significantly higher adsorption capacity for PFOA (139 mg/g) than GenX (11.8 mg/g), reflecting the greater efficacy of hydrophobic interactions in removing long-chain PFASs. The lower affinity of GenX was attributed to its shorter perfluoroalkyl chain and the presence of ether linkages, which diminish its interactions with the adsorbent surface. Kinetic modeling confirmed that the adsorption of both compounds followed pseudo-second-order kinetics, indicating a chemically controlled and stable adsorption process [30].

### Removal based on water quality conditions

#### pH

The pH of water significantly affects the elimination of PFASs by MOF-based adsorbents because it profoundly influences the surface charge of the MOF and the ionization state of the PFAS molecules [18]. The adsorptive removal of PFOS using A-CMOF was observed under different solution pH conditions (2–8) [26]. These results suggested that the most effective adsorption of the A-CMOF (nearly 100%) was obtained under acidic conditions (pH 3). This could be attributed to the protonation of –NH_2_ functional groups in acidic environments. Consequently, the positively charged –NH_2_ groups develop active sites for the –SO_3_^−^ headgroup of the PFOS species, promoting the adsorption activity. Nevertheless, at pH levels greater than 7, the presence of OH^−^ ions could compete with anionic PFOS molecules for the adsorption sites, accordingly exerting an unfavorable effect on the environment for PFOS removal. These results were consistent with those of a previous investigation on PFOS adsorption using aminated adsorbents, highlighting the significance of the initial pH on adsorption effectiveness [43].

**Fig. 1:**
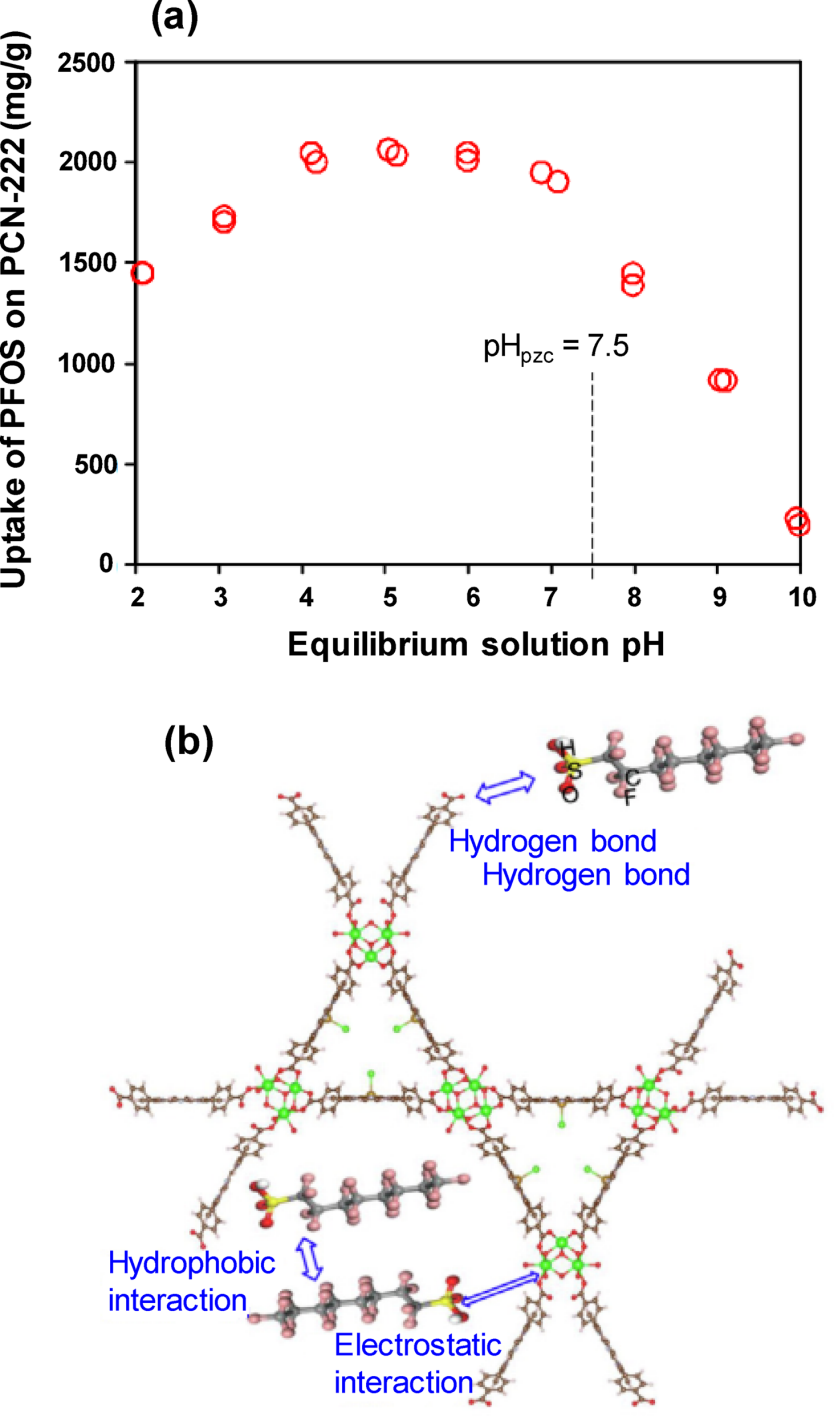
**a** PFOS removal by PCN-222 as affected by equilibrium solution pH under initial concentration of 500 mg/L and **b** schematic diagram of PFOS adsorption mechanism on PCN-222 [27]

Over a wide range of solution pH values (2–10), PCN-222 showed the highest PFOS adsorption capacity (approximately 2000 mg/g) at pH 5 (Fig. 1a) [27]. The adsorption effectiveness of PCN-222 MOF diminished as the solution pH exceeded 5, which was largely attributable to the extent of PFOS deprotonation during its interaction with PCN-222 and secondarily to the point of zero charge (PZC) of PCN-222. PFOS has a pKa of 3.27, indicating that it is predominantly deprotonated (negatively charged) in the pH range above 3.27 and remains anionic from pH 4 to 10. As the pH exceeds 5, the increasing concentration of OH⁻ can compete with anionic PFOS species for binding to the cationic Zr sites on the adsorbent surface, potentially reducing PFOS adsorption efficiency. This resulted in a reduction in the PFOS adsorption capability of PCN-222 when the solution pH exceeded 5. At pH < 5.0, the high concentration of H^+^ ions may have resulted in partial neutralization of PFOS anions, weakening the electrostatic attraction to the cationic sites on PCN-222 and resulting in lower adsorption [44]. The PZC of PCN-222 is 7.5 [44], suggesting that the MOF surface is positively charged (cationic) under the optimum pH conditions. Thus, at pH 5, the adsorption of PFOS onto PCN-222 is predominantly influenced by electrostatic interactions between the positively charged zirconium centers and the negatively charged PFOS species [27].

N-doped porous carbons prepared via the simple pyrolysis of ZIF-8 (denoted as N@ZIF-8) with tailored pore structures and surface functional groups were effectively utilized for the fast adsorption of PFOA [37]. In an acidic environment, the protonation of the O- and N-containing functional groups on the adsorbent surface results in a positively charged surface, which enhances the adsorption of negatively charged PFOA ions through electrostatic attraction. In contrast, at relatively high pH levels, the deprotonation of these functional groups results in a negatively charged surface, which repels anionic PFOA species [45]. At minimal ionic strength (nearly 0 mM NaNO_3_), the removal efficiency was consistently maintained at approximately 98% throughout the wide pH range (3.0–11.0). At increased ionic strength (100 mM NaNO_3_), a comparable pattern was achieved, with a marginal decrease in removal efficiency to 90%. The results indicate that electrostatic interactions are not the primary mechanism for PFAS adsorption onto N@ZIF-8, whereas other interactions, such as hydrophobic or π–π interactions, may be more significant [37].

#### Background ions

**Fig. 2:**
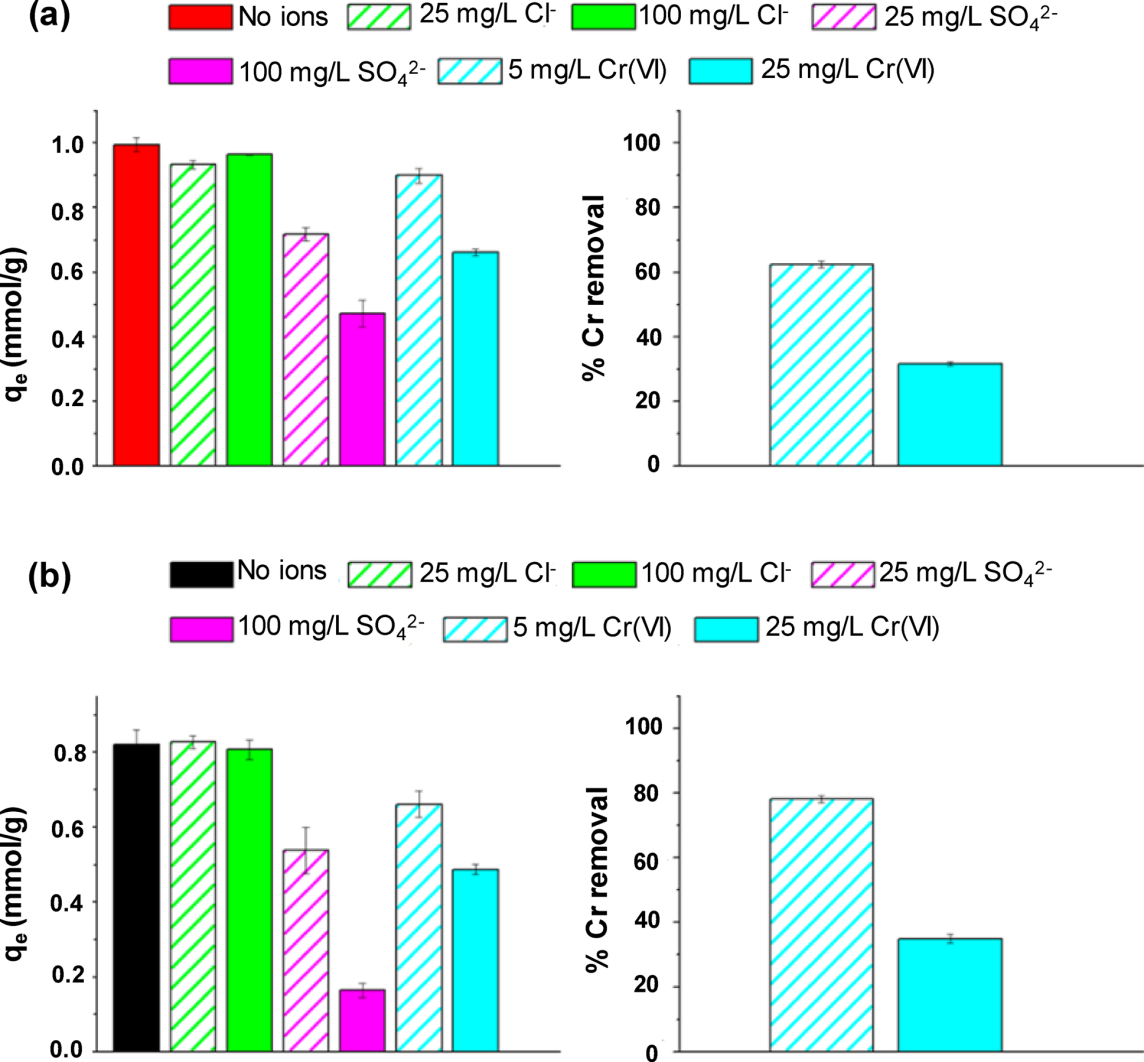
Effect of ions on PFOS adsorption at pH 3 and percent Cr(VI) removal on **a** UiO-66@10 and **b** UiO-66@25 [10]

The influence of background ions on PFAS removal by MOFs occurs primarily through competition, charge screening, ion bridging, and potential destabilization of the framework. Thus, examining these effects is crucial for measuring MOF performance under realistic environmental conditions [46]. Metal plating constitutes a significant industrial source of PFOS contamination, representing almost 50% of the industrial PFOS emissions in China in 2010 [47]. Consequently, the influence of coexisting ions on PFOS adsorption should be examined. Clark et al. investigated three predominant anions (Cl^−^, SO_4_^2−^, and Cr(VI)) often present in chromium plating wastewater and their effects on PFOA removal by UiO-66@X [10]. Without coexisting ions, both UiO-66@10 and UiO-66@25 demonstrated enhanced PFOS adsorption at pH 3 relative to that at pH 5 with an adsorbent loading of 500 mg/L (Fig. 2). Elevating the pH beyond 5 had a minimal impact on PFOS adsorption. Cl^−^ ions exerted negligible influence on PFOS absorption, even at concentrations reaching 100 mg/L, presumably due to pre-existing Cl⁻ within the MOF, either coordinated to Zr nodes or located within the pores. SO_4_^2−^ and Cr(VI) anions markedly impeded PFOS adsorption owing to competition for active sites. For UiO-66@10, PFOS removal was reduced by 28% and 52% with the addition of 25 and 100 mg/L SO_4_^2−^, respectively. The reductions for UiO-66@25 were more significant at 34% and 80%, respectively. These findings highlight the significance of electrostatic interactions in PFOS adsorption at an acidic pH, with co-anions competing for active binding sites. A comparable trend was observed for Cr(VI) at pH < 7 [48]. At a Cr(VI) concentration of 5 mg/L, the adsorption of PFOS by UiO-66@10 decreased by 10%, and 33% at 25 mg/L. UiO-66@25 exhibited enhanced sensitivity with decreases of 19% and 41%, respectively. This behavior is consistent with other data, indicating that UiO-66 is an excellent adsorbent for Cr(VI) [49]. At reduced Cr(VI) concentrations, both UiO-66@10 and UiO-66@25 eliminated over 60% of Cr(VI) and significant amounts of PFOS, suggesting that defective UiO-66 materials are capable of the concurrent removal of PFOS and Cr(VI) from electroplating effluents [10].

A zirconium-based MOF (NU-1000) was employed for the adsorption of three perfluorosulfonic acids (PFSAs, C4 − C8) and six perfluorinated carboxylic acids (PFCAs, C1−C9) from groundwater [31]. These groundwater samples classified as investigation-derived wastewater (IDW) exhibited 11 PFAS chemicals exceeding detection limits (approximately 20–260 μg/L), with conductivities varying from 22 to 26,300 μS/cm. NU-1000 demonstrated significant PFAS removal efficacy, with total removal rates of 75–98% and individual PFAS removal rates of 80–100%, reflecting robust selectivity in the presence of co-contaminants. Nonetheless, the short-chain PFASs in IDW 1 and 2 exhibited reduced removal effectiveness, which was attributed to the relatively high conductivity of these samples. This is presumably owing to competitive coordination between PFAS and high concentrations of inorganic salts (e.g., NO_3_^−^, SO_4_^2−^, Cl^−^, and F^−^) at the Zr_6_ nodes of NU-1000. Conversely, long-chain PFASs are less influenced by the prevailing hydrophobic interactions with the pyrene-based linkers of NU-1000. The kinetics of competitive adsorption remain incompletely understood because of the rapid equilibrium (on the order of seconds); however, the structural integrity of NU-1000 was preserved after exposure, as verified using powder X-ray diffraction (PXRD). These data illustrate the strong efficacy and specificity of NU-1000 for eliminating PFAS from contaminated groundwater [31]. In a separate study, at an electrolyte concentration of 10 mM, both anions (chloride, nitrate, carbonate, and sulfate) and cations (potassium, sodium, calcium, magnesium, and ferric ions) showed insignificant effects on the PFOA adsorption process using N@ZIF-8, as the efficacy of PFOA elimination consistently exceeded 92.9% [37].

#### Natural organic matter (NOM) and other organics

**Fig. 3:**
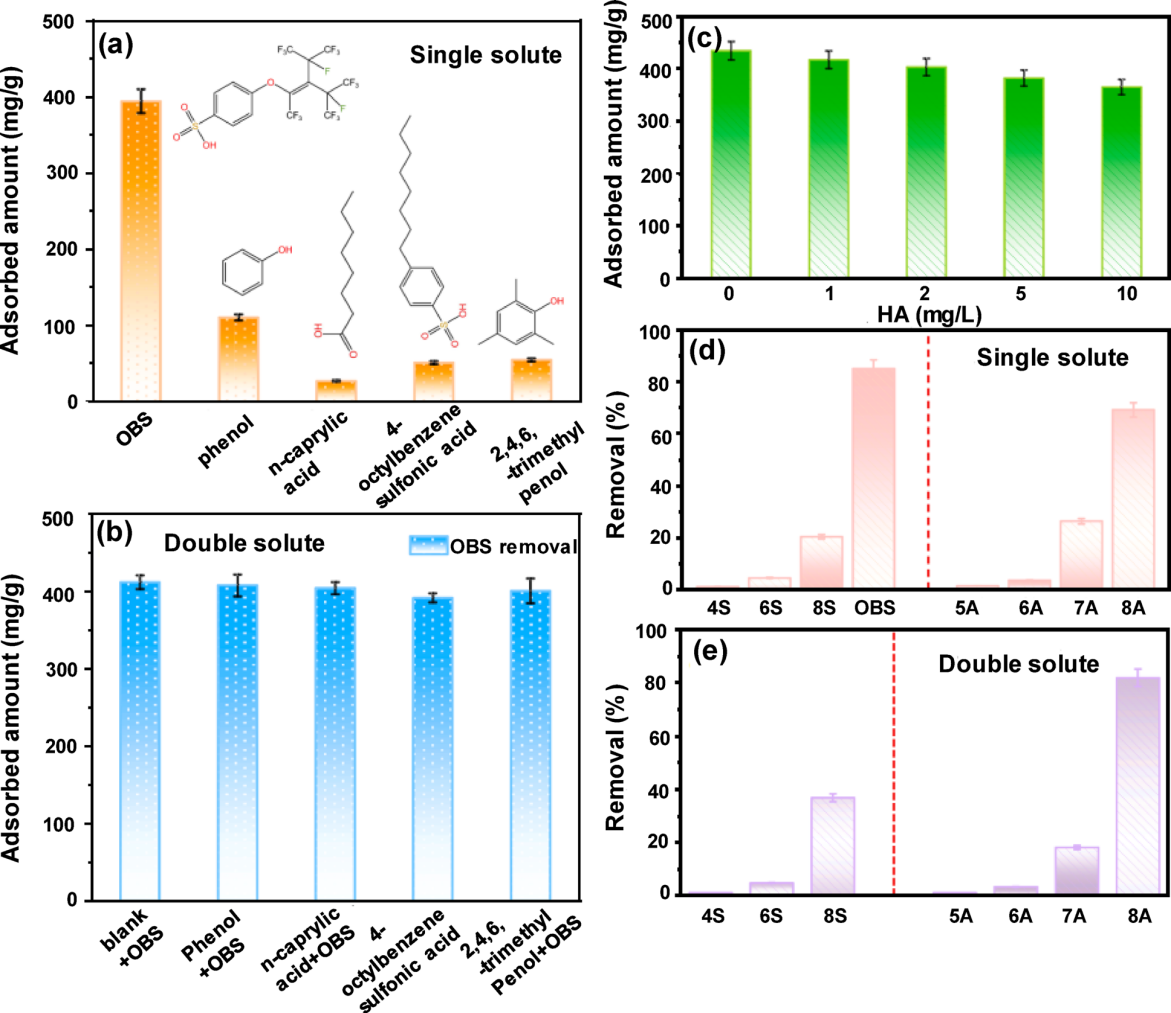
Selective adsorption of OBS onto the UiO-66-F4 in comparison with hydrocarbon organic compounds in **a** single-solute solution and **b** double-solute solution; **c** effects of HA on OBS removal by UiO-66-F4; adsorption of different PFAS by the UiO-66-F4 in **d** single-solute solution and **e** double-solute solution [28]

The presence of NOM and co-organic contaminants presents a challenge for PFAS adsorption by MOFs as they cause competitive adsorption, pore blockage, and surface fouling [20]. Jiang et al. evaluated the selective adsorption behavior of the fluorinated UiO-66-F_4_ MOF toward OBS and several PFASs, particularly in the presence of co-existing organic contaminants [28]. The MOF exhibited a markedly high adsorption capacity for OBS (394 mg/L), which was approximately 15 times greater than that of the hydrocarbon surfactant n-caprylic acid (27.2 mg/L), as shown in Fig. 3a. Although aromatic hydrocarbons such as phenol and trimethylphenol exhibited affinity owing to π–π interactions, their adsorption remained substantially smaller than that of OBS. This high selectivity was attributed to the fluorophilic and hydrophobic nature of UiO-66-F_4_ combined with its oleophobicity, which suppressed the adsorption of non-fluorinated oily substances [50]. When OBS was mixed with the four coexisting hydrocarbons, its adsorption decreased only slightly, indicating a strong resistance to competitive interference, and suggesting that OBS and hydrocarbons interact with UiO-66-F_4_ via different mechanisms (Fig. 3b). The presence of humic acid had a limited impact on the OBS adsorption, with only a approximately 10% decrease observed with 5 mg/L humic acid (Fig. 3c). The large size of humic acid likely prevented it from accessing the MOF pores, resulting in minimal competitive adsorption. However, humic acid may reduce the PFAS distribution in the solution by acting as a weak adsorbent [51]. The adsorption of sulfonic and carboxylic PFASs increased with C–F chain length in single and double solutions in the following order: perfluorobutanesulfonic acid < perfluorohexanesulfonate < PFOS (sulfonic acids); perfluoropentanoic acid < perfluorohexanoic acid < perfluoroheptanoic acid < PFOA (carboxylic acids), as shown in Fig. 3d and e. However, all PFASs showed lower adsorption than OBS, despite PFOS having the same number of fluorine atoms. The shorter, more compact structure of OBS facilitated pore diffusion, and the extra benzene ring enhanced the hydrophobicity and π–π interactions. In dual-solute systems, the presence of PFOS or PFOA enhanced OBS adsorption and vice versa. This was attributed to mixed micelle formation or multilayer adsorption on the MOF surface, suggesting a synergistic effect between the long-chain PFASs and OBS during adsorption [52].

Background organic contaminants are pervasive in groundwater and significantly affect PFAS adsorption efficacy [53]. The groundwater samples exhibited a wide range of total dissolved organic carbon concentration (0.11–268 mg/L) [30]. In particular, one of the groundwater samples containing the highest dissolved organic carbon concentration (268 mg/L), in contrast to 2.1 µg/L (total PFASs), exhibited a significantly decreased PFAS removal capability of approximately 30%. This unfavorable impact may be attributed to various mechanisms [54]: (1) NOM (e.g., humic acid) may compete with anionic PFAS for ion-exchange sites on MOFs [55]; (2) hydrophobic organic compounds could interact with MOF surfaces via hydrophobic interactions and H-bonding; (3) organic co-contaminants possessing strong basic functional groups may participate in acid–base interactions with the Zr_6_-node (a hard acid) of MOFs; and (4) macromolecular organic matter could have competition with PFASs for adsorption through electrostatic and/or hydrophobic interactions, thereby diminishing the availability of active sites [51]. In another study, humic acid was used to assess the effect of concurrent organic materials on the adsorption of PFOA by N@ZIF-8 [37]. Despite the high dose of humic acid (100 mg/L), the PFOA removal efficiency remained at 99.5%, demonstrating that humic acid had a negligible negative effect on the adsorption process. N@ZIF-8 exhibited significant resistance to interference by the water matrix composition, indicating its considerable potential for practical applications in the adsorption of PFOA from actual water systems.

The impact of the interaction with the cationic surfactant cetyltrimethylammonium bromide (CAB) on PFAS elimination using H@MIL-101(Fe) was examined systematically [42]. The complexation of CAB with perfluorobutanoic acid (C4) significantly enhanced the elimination efficiency of H@MIL-101(Fe) from 21.2 to 82.4% at a pH of 3. This improved adsorption continued at pH 9, indicating that the solid hydrophobic association between the CAB–perfluorobutanoic acid complex and H@MIL-101(Fe) exceeded the electrostatic repulsion between the anionic perfluorobutanoic acid molecules and the negatively charged MOF under alkaline conditions. In addition, the use of CAB significantly enhanced the adsorption of six mixed PFASs: PFOA (C8), perfluoroheptanoic acid (C7), and GenX (C6), with nearly 100% elimination within 30 min and over 90% elimination of perfluoropentanoic acid (C5). Increasing the CAB dosage to increase the PFAS complexation resulted in improved adsorption efficacy. Nonetheless, the total perfluorobutanoic acid (C4) was somewhat unaccomplished, presumably because of saturation of the adsorption sites. Furthermore, abundant CAB can compete for and occupy adsorption sites, thereby diminishing the efficacy of PFAS removal. For verification, the H@MIL-101(Fe) dosage was increased from 300 to 500 mg/L, and the original PFAS concentration was reduced to provide sufficient accessible adsorption sites. Under these conditions, the total elimination of all six PFASs was accomplished within 10 min at the natural pH of the surface water. Utilizing 10 mg/L CAB to facilitate the adsorption of 100 mg/L PFAS resulted in a residual CAB concentration of 1.2 mg/L with a removal effectiveness of 87.4%. Conversely, without PFASs, the residual CAB content decreased to 0.54 mg/L, and the removal efficiency increased to 94.6%. These results indicated that H@MIL-101(Fe) was effective for CAB adsorption, whereas the presence of PFASs reduced the number of accessible adsorption sites for CAB. Consequently, by moderating the dosage of H@MIL-101(Fe), simultaneous and total adsorption of both PFASs and CAB can be accomplished, thereby effectively mitigating secondary pollution [42].

## Removal mechanisms

### Experimental studies

The significance of understanding PFAS removal mechanisms using MOF-based adsorbents in experimental research is to enhance material design, optimize performance, and guarantee long-term applicability in practical water treatment [25]. In particular, identifying predominant adsorption mechanisms (e.g., electrostatic attraction, hydrophobic interaction, π–π stacking, and H-bonding) enables researchers to customize MOF structures such as linker chemistry, functional groups, and pore dimensions for enhanced PFAS removal efficiency [56].

PFOS adsorption onto PCN-222 is primarily governed by electrostatic interactions, hydrophobic effects, and H-bonding, as shown in Fig. 1b [27]. Electrostatic interactions were confirmed by varying the solution pH. The surface charge of PCN-222 varies with the pH owing to the protonation of the surface functional groups, which clearly affects the adsorption of anionic PFOS [57]. The positively charged Zr centers of PCN-222 (PZC = 7.5) strongly interact with the negatively charged head groups of PFOS (PZC = 3.27) [46], facilitating effective adsorption at the experimental pH of 5. PFOS forms micelles at concentrations above 100 mg/L, exceeding the critical micelle concentration [58], and further enhancing adsorption. For instance, at an original concentration of 200 mg/L, PCN-222 exhibited an adsorption capacity of 1690 mg/g. Future molecular simulations may help clarify the behavior of PFOS micelles during their interactions with MOF surfaces. The PFOS used in this study (C8 backbone, C–F chain length of 17) exhibited strong hydrophobicity. High PFOS concentrations (e.g., 500 mg/L) resulted in an extremely high adsorption capacity, suggesting significant hydrophobic interactions between the fluorinated alkyl tail and the nonpolar regions of PCN-222 [59]. The addition of tetrakis(4-carboxyphenyl)porphyrin as an organic linker may impart additional hydrophobicity to the MOF. PFOS sulfonate groups can form hydrogen bonds with coordinated water molecules inside MOF channels. In PCN-222, Zr_6_(μ-OH)_8_(OH)_8_(CO_2_)_8_ clusters provide hydroxyl groups that can interact with functional groups (e.g., –COOH) via H-bonding [60]. The presence of H-bonding was supported by Fourier-transform infrared spectroscopy (FTIR) spectral bands at 1603 and 1412 cm^−1^. The oxygen atoms of PFOS may act as hydrogen bond acceptors, forming bonds with the hydrogen atoms of the PCN-222 ligand framework [27].

Several sophisticated characterizations, such as FTIR, PXRD, and single-crystal XRD, were conducted to clarify the PFOA adsorption mechanisms in PCN-999 [36]. After PCN-999 was immersed in a 1000 mg/L PFOA solution for 3 d, FTIR analysis of the used PFOA@MOF exhibited new bands at approximately 1200 and 1140 cm^−1^, indicative of C–F stretching and C–C–C bending vibrations [61], thereby verifying successful PFOA loading. A notable shift of the C = O stretching vibration (~ 1604 cm⁻^1^) indicated Lewis acid–base interactions and coordination between the PFOA carboxylate groups and unsaturated Zr sites of the MOF. The PXRD analysis verified the structural property of PCN-999 upon adsorption. The porosity study indicated a significant reduction in pore volume and size, with only one prominent pore size of approximately 1.5 nm, implying mesopore hindrance by PFOA. Single-crystal XRD was conducted on the PFOA@MOF following a 10-d immersion to elucidate the binding mechanism. The structure maintained its *Cmmm* space group and exhibited a minor unit cell enlargement. Crystallographic findings indicated that PFOA molecules particularly coordinate with the biformate-bridged (Zr_6_)_2_ secondary building units, rather than the Zr_6_ clusters, underlining unique coordination behavior resulting from varying unsaturated binding environments [62]. Each (Zr_6_)_2_ secondary building unit coordinates with eight PFOA molecules, resulting in a total of 16 coordinated nodes. The PFOA molecules assume a zigzag shape within the mesopores, facilitated by spatial freedom. The experimental PFOA absorption (1090 mg/g or 3.14 PFOA/ligand) surpasses the crystallographically established value (1.00 PFOA/ligand), suggesting the influence of supplementary noncovalent physical adsorption [36].

### Theoretical studies

**Fig. 4:**
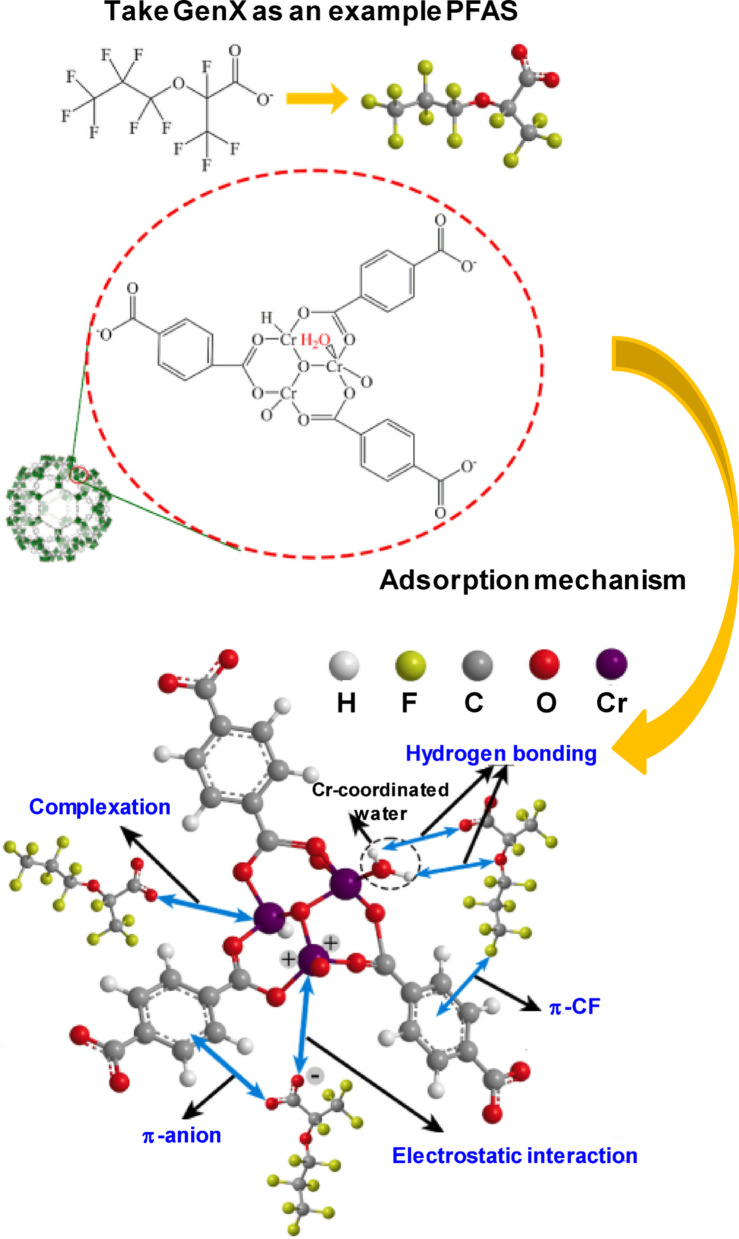
Schematic diagram of PFAS adsorption mechanism on MIL-101(Cr) [29]

Theoretical studies on PFAS removal using MOFs are important for enhancing experimental research, providing profound insights into molecular interactions, material design, and forecasting performance before synthesis [63]. Adsorption tests and material characterization suggest that the adsorption mechanisms of various PFASs onto MIL-101(Cr) likely include electrostatic interactions, complexation, H-bonding, π–CF interactions, and π–anion interactions (Fig. 4) [29], all influenced by binding energy related to electron cloud density [64]. The density of the electron cloud, which is indicative of the molecular electronegativity, is predominantly affected by the peripheral atoms (e.g., F, O, and H). Furthermore, the adsorption kinetics indicated that PFAS absorption is affected by diffusion constraints and steric hindrance, mostly depending on the chain length [63]. This study introduced a novel parameter, the average electronegativity of individual moieties (*Me*), which integrates the effects of electronegativity and molecular structure. *Me* was determined by summing the electronegativities of the peripheral atoms and dividing by the number of central atoms (C and S), which is indicative of the chain length. The *Me* values were ranked as follows: GenX > PFOS > perfluorohexanoic acid > PFOA > 6:2 fluorotelomer sulfonate, exhibiting strong positive correlations with both the adsorption capacities and adsorption coefficients at varying equilibrium concentrations. These results were further verified by the comparable tendencies observed for PFAS adsorption onto NU-1000 [31].

**Fig. 5:**
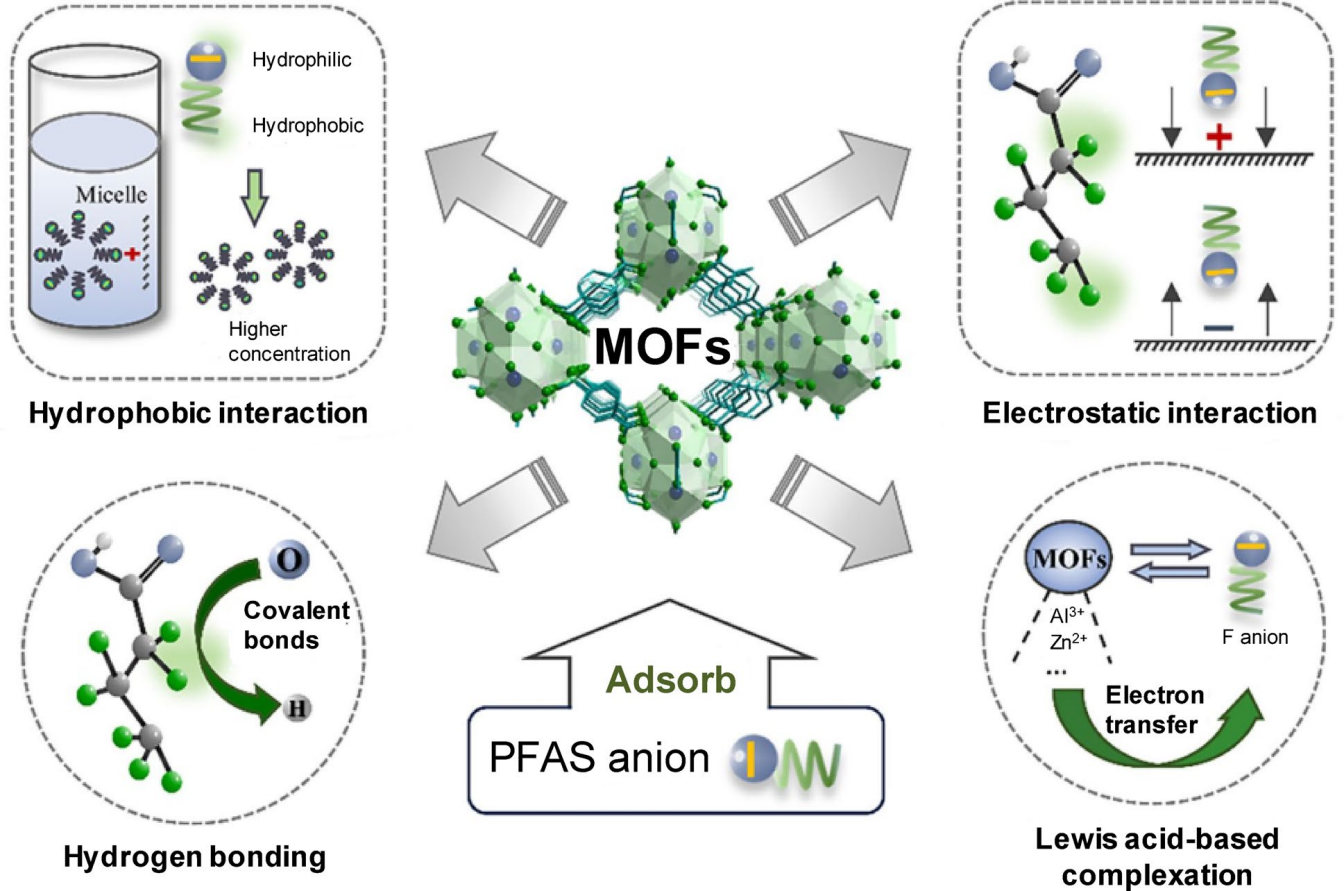
Adsorption mechanisms of PFAS by MOFs [18]

The examination of fluorocarbon and hydrocarbon interactions is crucial for understanding and enhancing the adsorptive removal of PFASs using NU-1000, as it elucidates the molecular mechanisms that govern selectivity, capacity, and efficiency [33]. Comparisons between the simulated adsorptive loading and theoretical packing based on molecular size and pore volume indicated that intermediate-sized analytes (i.e., four-carbon polar and three-carbon nonpolar species) achieved optimal packing inside NU-1000. These molecules appear to be sufficiently small to reach the adsorption sites adjacent to the metal nodes, but sufficiently large to establish many advantageous interactions. Variations in this optimal chain length in either direction resulted in diminished packing densities; however, moderate increases were noted for polar compounds with chain lengths of six to eight, potentially attributable to micelle-like aggregation within the mesopores, as inferred by analogy with the aggregation behavior of PFOA–poly(ethylene oxide) observed in a previous study [65]. In all simulations, an increase in the PFAS chain length resulted in a reduction in the number of adsorbate molecules per unit cell, presumably because of steric hindrance. Smaller molecules exhibited enhanced mobility and pore accessibility, facilitating increased packing density and improved interactions with the adsorption sites. The adsorption of alkanoic acids and PFAAs was determined to be thermodynamically advantageous both under dry conditions and in the presence of co-adsorbed water. Radial distribution functions demonstrated significant ordering of polar groups surrounding the Zr_6_ nodes [66], along with considerable alignment of the nonpolar termini of amphiphilic compounds. Nonetheless, nonpolar species showed no absorption in the presence of water [33]. Figure 5 describes key adsorption mechanisms of PFASs by MOFs. Table 1 summarizes the adsorptive removal of selected PFASs using MOF-based nanoadsorbents, as documented in the current study. For comparison, a summary of PFAS removal by graphene oxide (GO)-based nanoadsorbents is presented in Table 2.

**Table 1:** Summary of removal of PFASs by MOF-based nanoadsorbents

Adsorbent	Target PFAS*/C*_o_ (mg/L)	Experimental condition	*q*_m_ (mg/g)/ % removal	Main finding	References
Amine-Cu@MOF	PFOS/10–100	Time = 60 min pH 2–8, room temp synthetic water	670 at pH 3	According to the pseudo-second order kinetic model and Langmuir isotherm, PFOS was taken up by monolayer chemisorption	[26]
MIP-202	PFBS PFPeS PFBA PFPeA/ 0.005–100	Time = 1–30 min pH 2–9 room temp synthetic water	526–621	The PFAS-protonated amine moieties in MIP-202 structure were electrostatically interacting with Zr clusters, resulting in a complex adsorption mechanism	[67]
PCN-222	PFOS/ 50–500	Time = 0.25–24 h pH 2–11 temp. = 25 °C synthetic water	2257	A combination of electrostatic attraction, hydrophobic interaction, and hydrogen bond formation was responsible for the adsorption of PFOS on PCN-222	[27]
Defective UiO-66	PFOS PFBS/ 25–500	Time = 0–2 h pH 3–9 temp. = 25 °C synthetic water	1.24 mM/g (PFOS)	The increased internal surface area and the number of coordinatively unsaturated Zr sites, defective UiO-66 showed substantial adsorption capacities for both perfluoroalkyl sulfonates	[10]
MIL-101(Cr)	9 PFASs/ 0–2; 0.114	Time = 0–7 h pH 3–9 temp. = 25 °C groundwater	89.7–156 µM/g	Electrostatic interaction, complexation, H-bonding, π-CF interaction, and π-anion interaction are associated with the molecular electronegativity of individual PFASs	[29]
UiO-67-F_2_ UiO-67-NH_2_ UiO-68-(CF_3_)_2_ UiO-67	PFOA/ 1000	temp. = 0–20 °C synthetic water	928	The fluorinated UiO-67 analogue showed a high equilibrium PFOA uptake of 928 mg/g and 1,700 mg/g at an initial PFOA concentration of 1,000 and 3,200 mg/L, respectively	[38]
UiO-66 with various functional groups	PFOA PFOS GneX/ 0.002–0.1	Time = 0–3000 min temp. = 25 °C synthetic water	439–3631	The importance of guest-accessible porosity in decontaminating water from PFAS and other persistent organic compounds	[68]
Waste-derived MIL-101(Cr)	PFOA/ 10–50	Time = 0–30 min pH 2–9 temp. = 25 °C synthetic water	13.6–720	The PFOA adsorption capacity was the highest (720 mg/g), with the adsorption process taking less than 7 min	[39]
Fluorinated UiO-MOFs	OBS/ 1–250	Time = 0–70 h pH 7 temp. = 25 °C synthetic water	451	F-MOFs captured OBS molecules from the long distances via hydrophobic interaction, and then formed a F–F non-covalent interaction driven by a dispersive force to further attract OBS within the shorter distances	[28]
NU-1000	PFSA PFCA/ 10–50	Time = 30 min temp. = 25 °C synthetic/ground water	400–620 (PFSA) 201–604 (PFCA)	Long-chain PFAS compounds exhibited preferential adsorption over short-chain PFAS compounds	[31]
NU-1000 UiO-66 ZIF-9	21 PFASs/ 0.002–0.350	Time = 1 min-48 h temp. = 25 °C ground water	58–99% (NU-1000) 2–95% (UiO-66) < 10% (ZIF-9)	Electrostatic interactions between anionic PFAS and the cationic Zr_6_ metal node of NU-1000 are the dominant mechanism for anionic PFAS adsorption	[30]
PCN-999	PFOA/ 100–5000	Time = 0–12 h temp. = 25 °C synthetic	1089	PCN-999’s atomically precise structure, coupled with its outstanding chemical and physical stability, results in remarkable PFOA adsorption	[36]
TFA-MOF-808	PFOA/ 100–800	Time = 24 h temp. = 25 °C synthetic water	1581 2496	Enhancing performance of PFAS-MOFs through multiple interactions, including hydrophobicity and coordinative bonding	[40]
Heat treatment derivative MIL-101(Fe)	Several PFASs/ 0.1, 1.0	Time = 0–8 h pH 3–7 temp. = 25 °C synthetic/surface water	139 (PFOA) 11.8 (GenX)	At pH 7.0, six PFAS containing 4–8 carbon atoms could be completely coadsorbed in surface water within 10 min largely due to the hydrophobic interaction mechanism	[42]
N-doped porous carbons derived from ZIF-8	PFOA/ 1–200	Time = 0–240 min pH 3–11 temp. = 25 °C synthetic water	256/ 99.7%	The adsorption equilibrium of PFOA on N-doped porous carbons was achieved in 10 min with a removal efficiency up to 99.7%	[37]
UiO-66	Five PFASs/ 0.1–30	Time = 0–1,600 min pH 3–11 temp. = 25 °C synthetic water	> 95%	Both short-chain and long-chain targets exhibited different rate-controlling steps and interaction sites during adsorption	[69]

*C*_o_, initial concentration; *q*_*m*_, maximum adsorption capacity; temp., temperature; MIP-202, zirconium and L-aspartic acid-based MOF; PFBS, perfluorobutanesulfonic acid; PFPeS, perfluoropentanesulfonic acid; PFBA, perfluorobutanoic acid; PFPeA, perfluoropentanoic acid; OBS, sodium p‑perfluorous nonenoxybenzenesulfonate; PFSA, perfluorosulfonic acid; PFCA, perfluorinated carboxylic acid; NU-1000, zirconium-based MOF; PCN-999, unprecedented zirconium-based MOF; TFA, trifluoroacetic acid

**Table 2:** Summary of removal of PFASs by GO-based nanoadsorbents [70]

Adsorbent	Target PFAS*/C*_o_ (mg/L)	Experimental condition	*q*_m_ (mg/g)/% removal	Main finding	References
*Graphene*					
Graphene/ functionalized graphene	PFBS PFHxS PFOS/ 500	Time = 24 h temp. = 25 °C synthetic water	27.7% (PFOS)	As compared to short-chain PFAS, nanobubbles contributed more significantly to long-chain PFAS	[71]
*GO/rGO*					
Microfluidics-generated GO microspheres	PFOS/ 0.1	Time = 3 min pH 7 temp. = 25 °C synthetic water	98% within 2 min	As a result of the interaction between Mg^2+^ and PFOS molecules, the metal composite significantly enhanced PFOS removal efficiency	[72]
GO/ polyethyleneimine-GO	PFOA/ 10–100	Time = 15 h pH 4–10 temp. = 25 °C synthetic water	231	The formation of hemi-micelle or micelle structures might occur during PFOA adsorption at high concentrations	[73]
Cationic surfactant functionalized GO	PFOA/ 25–200	Time = 50 min pH 3.5 temp. = 298–318 K synthetic water	710/ 98.5%	Spectroscopic and zeta potential analyses confirm electrostatic and hydrophobic interactions and H bonding for adsorption	[74]
CAB-GO	11 PFASs/ 0.02	Time = 1 h temp. = 298–318 K river water	48.8/ almost 100%	PFAS removal effectiveness was not affected by pH, NOM concentration, or ionic strength, except for PFBA	[75]
Polyethyleneimine@F-GO	PFBA, PFBS, PFHxS, PFNA/ 250 ng/L each	Time = 4 h pH 7 temp. = 25 °C synthetic water	~ 220 (PFNA)/ 99%	In order for PFAS to be removed, fluorophilic, electrostatic, and hydrophobic interactions play a significant role	[76]
*Graphene/GO-based composites*					
Fe-graphene hydrogel	PFOA/ 10	Time = 12 h pH 4–8, room temp synthetic water	218/ 74–90%	An excellent removal performance was shown in five runs using Fe-rGO hydrogels at the various pH levels	[77]
La-Mn-Fe@GO	PFOA/ 0.5–3.0	Time = 5 – 1440 min temp. = 25 °C synthetic water	1.61/ ~ 90%	As a result of the addition of anions, cations, and other organic compounds, no notable differences were observed in the adsorption of PFOA	[78]
ZMF@rGO	PFOA, PFOS/ 10	Time = 12 h temp. = 25 °C synthetic water	9.06 (PFOA) 9.9 (PFOS)	The adsorption of PFOA and PFOS on the Zr-MnFe_2_O_4_@rGO nanohybrids was dominated primarily by electrostatic attraction and hydrophobic interaction	[79]
ZF-CB@rGO	PFOA, PFOS/ 10–50	Time = 900 min pH 3–10 temp. = 25 °C synthetic water	16.1 (PFOA) 21.6 (PFOS)	It has been determined that PFOA and PFOS are removed through electrostatic attractions and hydrophobic interactions	[80]
Cu-F@rGA	PFOA/ 0.5–10	Time = 12 h, pH 4–10 temp. = 25 °C synthetic water	25.8	The Cu-F@rGO_AG electrode is 489% more efficient at + 0.8 V than an open circuit electrode and 45.9% more efficient than an unmodified electrode at the same voltage	[81]
Magnetic amino-functionalized GO	PFOA, PFOS, PFBS, PFHxS/ 0.005–0.5	Time = 60 min pH 6.5 temp. = 25 °C synthetic water	95%, long-chain PFAS; 85%, short-chain PFAS	As a result of electrostatic interactions, amine groups are critical for PFAS adsorption, particularly short-chain PFAS	[82]
PVA@UiO-66-NH_2_/GO	PFOA/ 1–10	Time = 12 h pH 3–9 temp. = 25 °C synthetic water	9.9	PFOA removal efficiency was maintained over multiple cycles, and optimal reduction occurs at approximately pH 5	[83]
Amino@GO_AG	PFOA/ 10–1000	Time = 48 h pH 1.5–9.5 temp. = 25 °C synthetic water	1575/99.95%	PFOA adsorption kinetics and isotherms were well-fit using pseudo-second-order and Freundlich models	[84]
C_n_-βCD@GO	PFBA/ 0.5 µg/L	Time = 0.25–24 h temp. = 25 °C tap water	65%	It was demonstrated that GO and βCD can adsorb fluoroalkyl chains of different lengths in PFAS mixtures	[85]
Fe_3_O_4_@rGO	OBS/20	Time = 42 h pH 7 temp. = 25 °C synthetic water	362 μM/g	Electrostatic, π-π and hydrogen bonding interactions played important roles in OBS adsorption, and the quaternary N in Fe_3_O_4_/rGO was a critical adsorption site	[86]
TiO_2_ quantum dots@sulfonated graphene	PFOA/5 (0.0012 mM)	Time = 10 h pH 7 temp. = 25 °C synthetic water	0.0632 mM/g	There were large amounts of PFOA reserved in the sulfonated graphene aerogel, resembling a reservoir	[87]

*C*_o_, initial concentration; *q*_*m*_, maximum adsorption capacity; rGO, reduced GO; temp., temperature; ZF, zinc ferrite; ZMF, Zr-MnFe_2_O_4_; CB, chitosan biopolymeric spheres; PVA, polyvinyl alcohol; PFBS, perfluorobutanesulfonic acid; PFHxS, perfluorohexanesulfonate; AG, amino-functionalized graphene oxide; CAB, cetyltrimethylammonium bromide; PFBA, perfluorobutanoic acid; PFNA, perfluorononanoic acid; OBS, sodium *p*-perfluorous nonenoxybenzene sulfonate

## Reusability and recovery of MOF-based nanoadsorbents

The reusability and recovery of MOF-based nanoadsorbents for PFAS removal are essential for converting laboratory results into practical and scalable water treatment solutions [20]. The possible reuse of the fabricated A-CMOF was assessed by conducting adsorption/desorption experiments [26]. To assess the water resistances of the Cu-based MOF and A-CMOF, 100 mg of each adsorbent was suspended in 50 mL of deionized water at pH 7. The suspensions were preserved under ambient conditions for 4 and 7 d, respectively. After each exposure period, the samples were analyzed to evaluate structural alterations. Additionally, the PFOS adsorption capacity of the materials was assessed before and after 7-d water exposure to evaluate the impact of extended water contact on their adsorption efficacy. The results confirmed a negligible reduction in the PFOS removal efficiency following recovery of the adsorbent, either due to the saturation of effective adsorption sites or the chemical properties of the adsorption process. The A-CMOF demonstrated a satisfactory capacity for reuse, maintaining approximately 93% of its initial PFOS adsorption capacity across eight consecutive cycles. This suggests that A-CMOF may be a viable alternative for practical water treatment applications, particularly in scenarios requiring repeated use [26]. In a separate study, MIL-101(Cr) exhibited remarkable regeneration efficacy, retaining nearly 80% of its PFAS adsorption capacity during three successive recovery and reuse cycles in surface water [29]. The MOF demonstrated significant structural stability, as evidenced by negligible Cr leaching, which remained below the detection threshold, as determined by inductively coupled plasma-optical emission spectrometry. These results demonstrated the excellent adsorption efficiency and recyclability of MIL-101(Cr) for various PFASs in surface water, emphasizing its potential as an effective sorbent for treating PFAS-contaminated water.

The regeneration efficacy of UiO-66-F_4_ was assessed during five adsorption–desorption runs using methanol (90%) as the regenerant [28]. In each run, UiO-66-F_4_ was immersed in a 100 mg/L OBS solution for 24 h, followed by filtration and desorption in methanol with agitation. The OBS concentration of the methanol solution was examined, and the regenerated adsorbent was filtered, dried, and utilized in the subsequent run. Methanol efficiently desorbed OBS with a consistent regeneration efficiency between approximately 85% and 95%, exhibiting minimal change among runs. The adsorption capacity was consistently measured at approximately 400 mg/g after five reuse runs, demonstrating significant reusability. The efficacy of methanol as a regenerant highlights the significance of hydrophobic interactions in OBS adsorption. Moreover, PXRD investigations after five runs showed no significant structural alterations in UiO-66-F_4_, confirming its remarkable structural stability and recyclability under aqueous conditions [28].

**Fig. 6:**
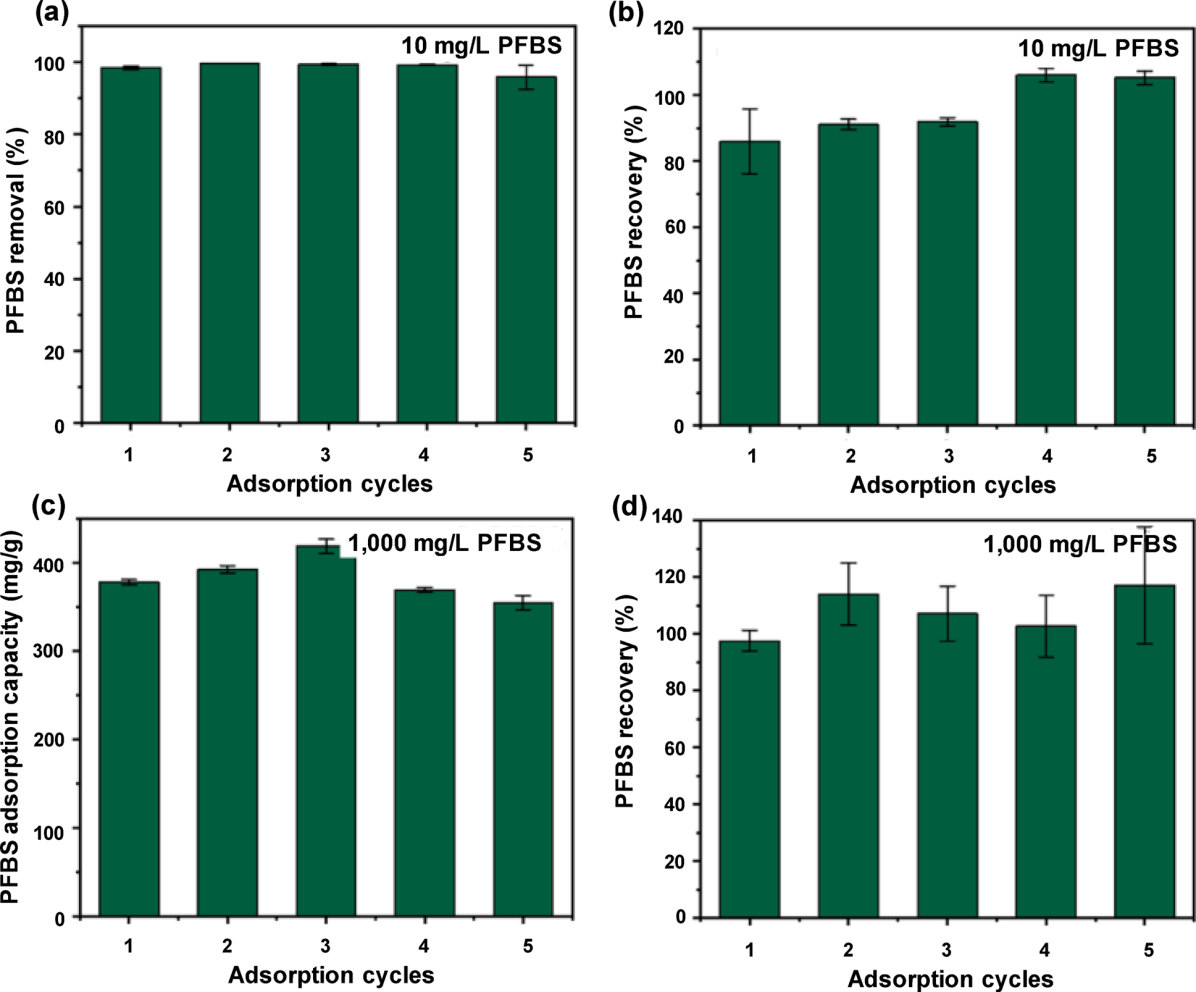
Regeneration characteristics of PFBS@NU-1000: **a**, **c** PFBS removal and **b**, **d** PFBS recovery rates of NU-1000 obtained from five successive adsorption and regeneration cycles using PFBS concentrations of (**a**, **b**) 10 mg/L and (**c**, **d**) 1000 mg/L, respectively [31]

The regenerative capacity of NU-1000 was assessed after perfluorobutane sulfonic acid (PFBS) adsorption using diverse combinations of inorganic salts and solvents [31]. The preliminary assessment indicated that pure MeOH and 0.1 M HCl in water exhibited restricted desorption efficacy. A 30:70 (v/v) mixture of 0.1 M HCl in MeOH yielded optimal PFBS recovery and was employed in systematic regeneration tests. Five successive adsorption–desorption runs were conducted using PFBS concentrations of 10 and 10,00 mg/L. The findings revealed remarkable regenerative efficacy: 96–100% elimination and 86–105% recovery during five runs for 10 mg/L perfluorobutane sulfonic acid (Fig. 6). In addition, adsorption capacities ranging from 355 to 419 mg/g were achieved with a recovery rate of 98–117%, aligning with the highest absorption of NU-1000 for 10,00 mg/L perfluorobutane sulfonic acid. The PXRD investigation verified the structural integrity after running, with no loss of Bragg peaks and only minimal broadening, indicating a small reduction in the crystallite size owing to tumbling. The Brunauer–Emmett–Teller (BET) surface area decreased by approximately 4% after one run and 6% after five runs, indicating negligible structural degradation. ^1^H nuclear magnetic resonance spectroscopy was performed on base-digested NU-1000 samples to examine the regeneration mechanism. In contrast to a previous study suggesting methoxy group coordination following MeOH exposure [52], no such coordination was observed. This indicates that Cl⁻ ions from the HCl/MeOH mixture probably displace PFBS at the Zr_6_ nodes, aligning with Lewis acid–base theory, which suggests that Cl⁻ exhibits better binding to Zr than methoxy or sulfonate groups [31].

After demonstrating the outstanding PFAS removal efficacy of TA@MOF-808, its applicability was assessed for water decontamination, emphasizing its regeneration and recyclability [40]. Conventional MOF regeneration techniques generally use acidic, basic, or organic solvents to eliminate the adsorbed contaminants [23]. However, for TA@MOF-808, such regeneration methods could restore the MOF to its original state owing to ligand exchange initiated during the initial adsorption run. To address this limitation, a direct regeneration method was developed by reimplementing the original TA functionalization mechanism. The introduction of the PFOA-loaded MOF into a solution containing excess TA facilitated the reverse ligand exchange, successfully rebuilding the TA-decorated framework in a single step with no apparent loss in crystallinity or structural integrity. This regeneration method facilitated the reuse of the MOF for a minimum of three successive PFOA adsorption runs, consistently achieving a nearly 100% removal efficiency. These results confirm the regeneration capabilities of TA@MOF-808 and validate the efficacy of the post-synthetic functionalization technique. Nevertheless, certain constraints persist for industrial-scale implementation, such as the possible release of ecologically unfavorable TA during ligand exchange [40].

Overall, MOFs exhibit variable chemical stability. While Cr- and Zr-based MOFs demonstrate notable water stability, the leaching of components remains a concern, although certain MOFs exhibit negligible Cr leaching below detection thresholds. Recyclability is similarly variable; some MOF-based nanoadsorbents maintain > 90% efficiency over multiple cycles. However, performance loss due to structural degradation or pore blockage can occur, limiting long-term effectiveness in some cases. Table 3 summarizes the experimental parameters related to the regeneration using MOF-based nanoadsorbents.

**Table 3:** Summary of regeneration methods of used MOF-based nanoadsorbents

Adsorbent	Regenerant	Experimental condition	Main finding	References
MIP-202	60% methanol and 40% 0.1 M HCl	Agitated overnight and centrifugation	Even after 8 runs, the adsorption capacity of all four PFAS showed only a negligeable reduction	[67]
MIL-101(Cr)	Methanol solution containing 1.25% NaNO_3_, 1.25% NaCl, 1.25% Na_2_SO_4_ and 1.25% Na_2_CO_3_	4 h shaking, centrifuged at 4500 rpm, freeze-dried at −50℃ for 8 h	Initial desorption efficiency of PFASs using the elution solution was generally 86.7%, while decreased slightly with time when MIL-101(Cr) was regenerated	[29]
UiO-66 with various functional groups	MeOH-NaCl	0.005 mg/L; PFOA, PFOS, GenX; microscale adsorption columns	PFOS and PFOA desorbed most readily from all four sorbents, with > 90% (PFOS) and > 80% (PFOA) of the adsorbed PFASs accounted for in the regeneration solvent	[68]
Fluorinated UiO-MOFs	MeOH (90%)	OBS 100 mg/L 2 h shaking at 170 rpm	Despite repeated reuse cycles, methanol was effective in desorbing OBS, and regeneration efficiency (approximately 85–95%) remained unchanged	[28]
NU-1000	30/70 v/v 0.1 M HCl/methanol	10/1000 mg/L PFSA, PFCA; centrifuged at 6000 rpm and filtered through a 0.25 µm nylon membrane	In samples with 10 mg/L PFBS concentrations, removal capacities of 96 to 100% over five regeneration cycles were observed, as well as recovery rates of 86 to 105%	[31]
TFA-MOF-808	MeOH	100 mg/L PFOA heated at 70 °C, vacuum for 1 h	The material could be reused for at least 3 PFOA adsorption runs, retaining complete (nearly 100%) removal efficiency	[40]
Heat treatment derivative MIL-101(Fe)	MeOH	1 mg/L PFOA 0.22 µm polyether sulfone membrane	Although four regeneration cycles were performed on H:MIL-101(Fe), it still displayed excellent adsorption performance, a stable structure, and rapid magnetic separation	[42]
N-doped porous carbons derived from ZIF-8	70% methanol (v/v) and 1% NaCl (w/w)	10 mg/L PFOA	After five runs, PFOA removal efficiency somewhat decreased from 99.4 to 85.9%	[37]
UiO-66	Methanolic ammonium acetate (400 mM)	Five PFASs, onicated for 30 min, centrifugation at 8000 rpm, vacuum drying at 100 °C for 2 h	Amorphous UiO-66 with the highest defect concentration showed a 4% decrease in adsorption efficiency, whereas crystalline UiO-66 showed an 8% decrease	[69]

## Conclusions and future perspectives

This review highlights the considerable potential of MOFs as advanced nanoadsorbents for the removal of PFASs, particularly PFOA and PFOS, from aqueous environments. This review emphasizes that both MOF characteristics (pore size, surface charge, and functional groups) and PFAS properties (chain length, functional moieties, and molecular charge) influence the adsorption performance. Among the MOFs assessed, NU-1000, PCN-999, UiO-66, and the TA-functionalized MOF-808 exhibited remarkable adsorption capacities and selectivities for both long- and short-chain PFASs, achieving very high removal in several cases. Operational variables, including pH, ionic strength, PFAS concentration, and background water quality, significantly influenced adsorption efficiency. In particular, MOFs with fluorinated or amine-functionalized ligands exhibit improved performance under acidic to neutral conditions, where electrostatic attraction and hydrophobic interactions dominate. Although background anions, NOM, and coexisting contaminants can interfere with adsorption, several MOFs, such as N@ZIF-8 and UiO-66-F4, exhibit strong resistance to fouling and competitive adsorption in the presence of NOM and coexisting contaminants. Mechanistic insights from experimental and theoretical studies reveal that PFAS uptake involves a combination of electrostatic attraction, Lewis acid–base interaction, hydrophobic effects, π–π stacking, and H-bonding. Advanced characterization techniques (e.g., FTIR, PXRD, and SCXRD) and simulation tools (e.g., Monte Carlo and density functional theory) are essential for elucidating the binding mechanisms and guiding rational MOF design. The regeneration and reusability of MOFs, which are crucial for real-world applications, have been demonstrated in several studies. Several MOFs, including A-CMOF, MIL-101(Cr), and TA@MOF-808, maintained high removal efficiencies over repeated adsorption–desorption cycles, validating their potential for practical deployment in PFAS-contaminated water treatment.

**Fig. 7:**
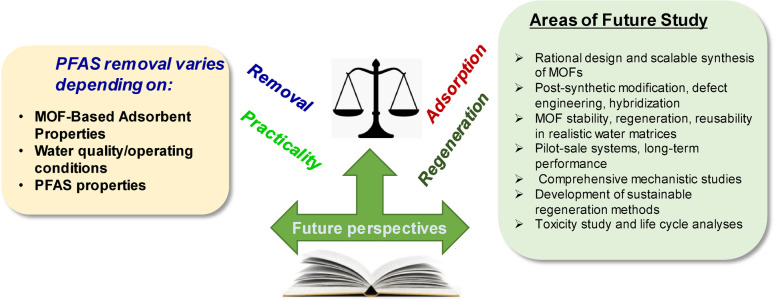
Future perspectives on MOF-based nanoadsorbents, water quality & operating conditions, and PFAS physicochemical properties for removing various PFASs

For enhanced practical applicability of MOF-based nanoadsorbents for PFAS remediation, several critical research gaps must be addressed despite the significant progress achieved in recent years. In particular, future research directions should be prioritized, which include (1) design of MOFs selective for short-chain PFASs because current MOFs often exhibit reduced effectiveness for these compounds, (2) computational screening for new ligands to accelerate the discovery of MOFs with improved selectivity and capacity, (3) life-cycle assessment for environmental safety to access the environmental impacts and toxicity of MOFs, and (4) policy-relevant standardization of testing protocols to generate comparable and policy-relevant performance data under realistic water matrices. Future efforts should focus on the rational design and scalable synthesis of MOFs with tailored surface functionalities, such as fluorophilic or amine-rich groups, to improve their selectivity and adsorption capacity across a broader spectrum of PFASs, including ultra-short-chain and emerging compounds, such as GenX and OBS. Techniques such as post-synthetic modification, defect engineering, hybridization with materials such as metal oxides or carbon-based supports, adsorbent-membrane hybrid systems, and catalytic degradation post-adsorption offer promising routes for enhancing the performance of MOFs under complex environmental conditions. To facilitate their real-world implementation, the stability, regeneration, and reusability of MOFs in realistic water matrices containing NOM, competing ions, and varying pH levels must be evaluated. Such assessments should be guided by standardized protocols and extended to pilot-scale systems, as the long-term performance under field-like conditions remains insufficiently understood. Furthermore, although the adsorption mechanisms of certain MOFs and PFASs have been partially elucidated, comprehensive mechanistic studies based on in situ spectroscopy, molecular simulations, and electron density mapping are required, particularly for short-chain PFASs. Another key challenge is the development of sustainable MOF regeneration methods. Although solvent-based regeneration is commonly employed in laboratory settings, it may be impractical on a large scale owing to environmental and economic limitations. Thus, future research should explore greener approaches, such as electrochemical, thermal, or ligand-exchange-based regeneration, along with closed-loop recovery systems. Additionally, concerns regarding ligand leaching, metal ion release, and potential secondary pollution must be rigorously assessed through toxicity testing and life cycle analyses. Ultimately, bridging the gap between laboratory development and practical deployment requires interdisciplinary collaboration between materials scientists, environmental engineers, toxicologists, and regulatory stakeholders. Aligning material innovation with real-world treatment goals and regulatory frameworks is crucial for advancing MOF-based nanoadsorbents from promising laboratory tools to effective and scalable solutions for PFAS-contaminated water remediation. Overall, Fig. 7 presents key considerations and future challenges associated with PFAS removal using MOF-based nanoadsorbents.

## Data Availability

The datasets used and/or analyzed during the current study are available from the corresponding author on reasonable request.
